# Complex structural variant visualization with SVTopo

**DOI:** 10.1186/s12864-025-12088-6

**Published:** 2025-10-09

**Authors:** Jonathan R. Belyeu, William J. Rowell, Juniper A. Lake, James Matthew Holt, Zev Kronenberg, Christopher T. Saunders, Michael A. Eberle

**Affiliations:** Computational Biology, PacBio, 305 O’Brien Drive, Menlo Park, CA 94025 USA

**Keywords:** Structural variant, Genome visualization, Long-read sequencing, Complex SV

## Abstract

**Background:**

Structural variants are genomic variants that impact at least 50 nucleotides. Structural variants can play major roles in diversity and human health. Many structural variants are difficult to interpret and understand with existing visualization tools, especially when comprised of inverted sequences or multiple breakend pairs.

**Results:**

We present SVTopo, a tool to visualize germline structural variants with supporting evidence from high-accuracy long reads in easily understood figures. We include examples of 101 visually complex structural variants from seven unrelated human genomes, manually assigned to ten categories. These demonstrate a broad spectrum of rearrangement and showcase the frequency of complex structural variants in human genomes.

**Conclusions:**

SVTopo shows breakpoint evidence in ways that aid reasoning about the impact of multi-breakpoint rearrangements. The images created aid human reasoning about the result of structural variation on gene and regulatory regions.

**Supplementary Information:**

The online version contains supplementary material available at 10.1186/s12864-025-12088-6.

## Background

Structural variants (SVs) are responsible for more base pairs of genomic variation than any other class of variant and play major roles in diversity, rare disease, and complex disease risk [1–3]. SV research has progressed in recent years from a focus on very large variations in copy number [4, 5] to much more inclusive studies [6–8] that report deletions, duplications, translocations, insertions, and inversions as small as 50 bp. SVs that do not precisely conform to any of these definitions have been reported as complex SVs [1, 2, 9–11]. Here we will define complex SVs as networks of two or more connected genomic breaks in a sample haplotype, excluding balanced inversions. These generally consist of combinations of rearrangements of multiple classic SVs, such as a duplication followed immediately by a deletion or an inverted duplication. A common type is the unbalanced inversion, where an inversion occurs in tandem with deletion or duplication of flanking genomic material, found to represent one fifth of inversions larger than 2 kbp [12].

Technologies such as optical mapping, nanopore sequencing, and single-molecule real-time sequencing that assay multi-kilobase fragment lengths can span multiple breakpoints in many SVs. New software tools for SV calling [13–17] and genome phasing [18, 19] use this additional information to increase SV recall and identify relationships between breakpoints in complex SVs. However, these improvements in identification of complex SVs create distinct new challenges in comprehending and annotating potential impacts on gene function or regulatory element structure.

Visualization is a critical step in variant assessment, and several visualization tools exist that allow researchers to evaluate likely functional impact at read-level resolution [20–22]. However, these short-read oriented visualizations struggle to represent the more complicated variation structures described by long reads in easily understandable ways. The challenges in comprehending SVs identified from long reads have resulted in development of new tools for SV visual curation, differentiating real variants from false positives by visual inspection of supporting evidence [23, 24]. Tools for improved comprehension and reasoning about SVs have also been created, often with a focus on extremely large-scale variation such as is seen in tumor contexts [25, 26]. Despite the development of these tools there remains a gap for visualizing complex SVs, likely leading to missed insights of these variants on human health.

SVTopo was created to simplify and enhance visual inspection of complex SVs in the germline, by helping humans comprehend effects on genome structure. It presents the essential read alignment components of complex SV evidence with up to twenty individual rearranged genomic blocks, creating clear and high-resolution graphical representations of complex rearrangements. SVTopo creates images that can aid human reasoning about complex SVs, improving understanding of genomic topography and the potential impacts on genomic health, gene structure, and regulatory elements.

In this study we demonstrate how SVTopo enhances analysis for many types of challenging variants, including multi-breakpoint SVs, inter- or intra-chromosomal translocations, inversions (with or without flanking insertions/deletions), and non-tandem inversions or duplications. SVTopo is readily added to genome analysis pipelines for small or large sequencing cohorts, greatly improving the accessibility of complex SV evaluation.

## Implementation

### Defining the structure of complex variation with SVTopo

**Fig. 1:**
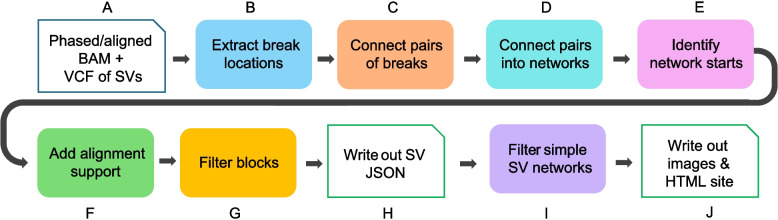
Visualization of complex SVs with SVTopo. **A** SVTopo runs on phased and aligned BAM files of long reads together with breakpoint information provided in an SV VCF file. **B** Break locations are extracted from SV call coordinates. **C** Read alignments and phase blocks are used to connect break locations together, forming pairs of connected breaks. **D** Pairs of breaks are further connected into networks of break locations, forming full rearrangement representations. **E** Start breaks are identified for each network of connected break locations, allowing the identification of the correct event order in the sample. **F** The number of supporting alignments for each pair of connected breaks in complete SV events is counted and added. **G** Filters eliminate regions with abnormal coverage or highly repetitive sequence. **H** Results are stored in JSON format, with additional annotations in BED format. **I** Simple SV networks, defined as those with only one or two same-chromosome, forward-oriented breaks, are filtered out (this optional filter removes the most common deletion and duplication variants). **J** Descriptive SV images are generated for complex SV networks, with an HTML website for table-based image browsing

SVTopo takes SV calls represented in a variant call format (VCF) file, as produced by most SV callers, and combines those calls with the aligned reads to provide a visualization of the evidence supporting the local structural variation (see Fig. 1). SVTopo connects genomic break locations described in the VCF file by using phased or spanning long-read alignments that support two or more breaks. Alignments are considered to support an SV break location if the alignment contains significant soft-clipping within 10 bp of an SV coordinate. If no alignments meet that criterion, the break coordinate is skipped. SVTopo creates full SV networks by connecting sets of coordinate pairs; these networks describe regions that are rearranged relative to the reference genome.

The final rearranged blocks are annotated with supporting alignment counts. Regions with abnormal coverage or mapping quality are pruned prior to providing visualization output for complex SV networks. The resulting SV information is written to file on a per-sample basis and used to inform automated generation of descriptive SV plots. A final (optional) filtering step during plot generation filters out SVs with one to two same-chromosome breaks unless an inverted block appears within the network. This filter removes most simple deletions, duplications, and unresolved single-ended BND calls, focusing SVTopo review on visually complex SVs. Finally, a table browser in HTML webpage format is generated for one or more samples processed together, with tools for variant search, regional prioritization, and image sharing. For more algorithm details, see Supplementary Note 1.

**Fig. 2:**
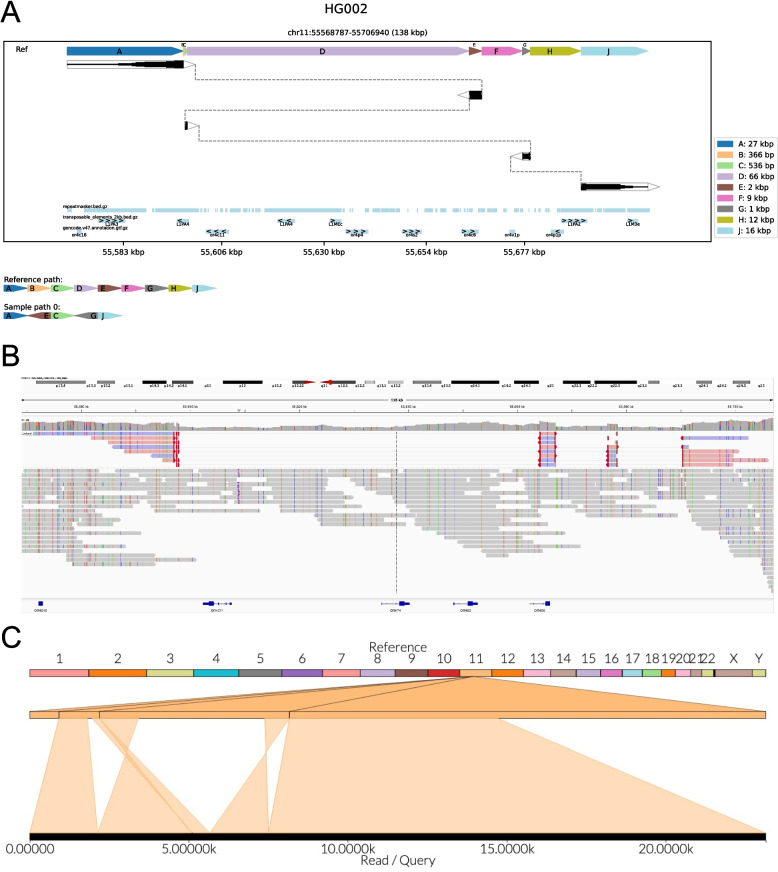
Visualization of a complex SV: Visual representations of a complex rearrangement, consisting of deletions of four component blocks and inversions of two. **A** An SVTopo plot clearly shows the order and orientation of inverted segments. Dark blocks represent alignment segments, made thicker where more alignment support occurs. Dotted lines connect blocks that are present in the sample. Blocks are ordered top-to-bottom as they appear in the SV haplotype and left-to-right as they appear in the reference genome. The bottom track below the main SV window contains a chain-plot of blocks in reference order, then in sample order and orientation. Blocks B, D, F, and H in the SV haplotype are eliminated, while C, E, and G are reordered. **B** IGV representation of the region. **C** Ribbon representation of the region

SVTopo images provide a simple comparison between the SV haplotype and reference structure. For example, in Fig. 2, an SV with a structure difficult to understand via IGV or Ribbon visualization is intuitively represented by SVTopo. SVTopo can optionally annotate images with gene locations, repeats, or other genomic features chosen by the user. Annotations may optionally include labels for orientation and region name. The image design carefully balances between simplicity and inclusion of essential details for complex SV interpretation, to enhance human understanding of SV structure.

## Results

### Multi-sample SV characterization

SVTopo was used to visualize SVs in a set of seven unrelated HiFi genomes: Genome in a Bottle Ashkenazi Trio Son HG002 [27] and six publicly released genomes from the three-generational Platinum Pedigree cohort [28]. These six genomes (NA12889, NA12890, NA12891, NA12892, 20,080, and 20,100) are the unrelated parental genomes in that cohort, thus representing all included ancestral haplotypes in the Platinum Pedigree. All genomes were aligned to the GRCh38 human reference and phased with WhatsHap v1.4. SV calling was performed with Sawfish v0.12.7. Sequencing coverage was approximately 30 × for HG002 and 200,080, 45 × for 200,100 and NA12890, and 60 × for NA12889 and NA12892. SVTopo generated SV images for all samples on a single processor in 8 h, 39 min, about 1.25 h per genome. The maximum RAM utilization was 4.75 GB.

**Fig. 3:**
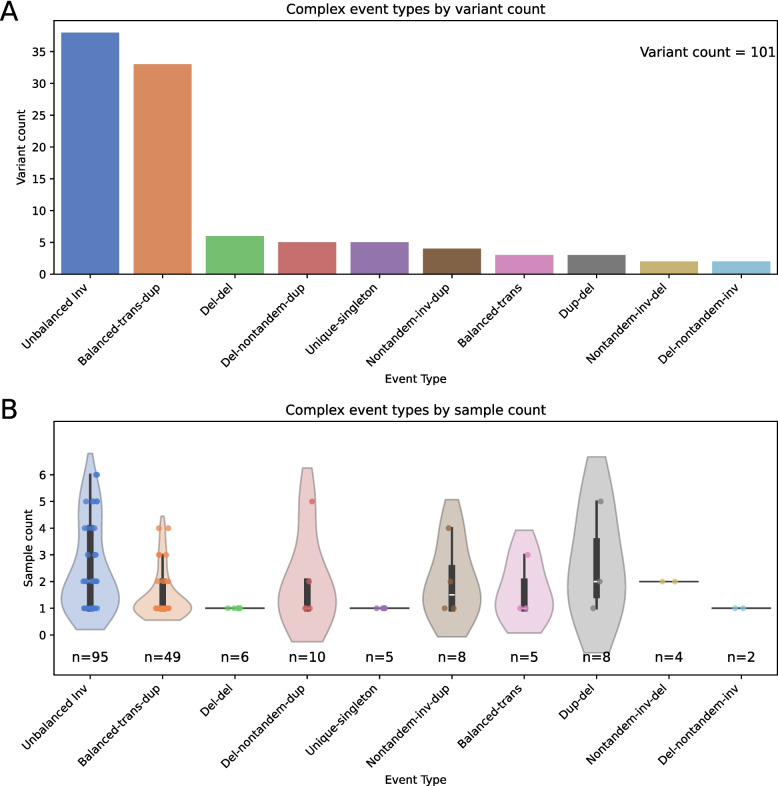
Types and distributions of complex SVs in seven unrelated genomes: 101 unique complex SVs were manually assigned to ten categories corresponding to variations on simple SV types, with five complex SVs grouped as Unique-singleton. **A** Counts of unique SVs assigned to each category. **B** Distributions of sample counts for variants belonging to each SV category

Excluding simple SV networks (omitted by default filtering at the image generation step, see Fig. 1), this analysis resulted in 446 images. 125 images were generated with incompletely resolved structure, many of which are indicative of alignment artifacts and false-positive SV calls (see Supplementary Figs. 1–5). 92 images represented canonical inversions, while 37 represented simple pairs of deletion or duplication variants associated by phasing. The remaining 192 images represented high-confidence complex SVs. Many of these rearrangements were shared by multiple samples, with a total of 101 unique complex SV loci [29]. While well-defined categories do not exist for most complex SVs, these variants were manually assigned to ten complex SV groups (see Fig. 3 and Supplementary Table 1), which we created for this variant set to simplify analysis. Five singleton SVs were individually unique and were grouped together as Unique-singleton variants, with the other nine groups containing structurally similar SVs.

**Fig. 4:**
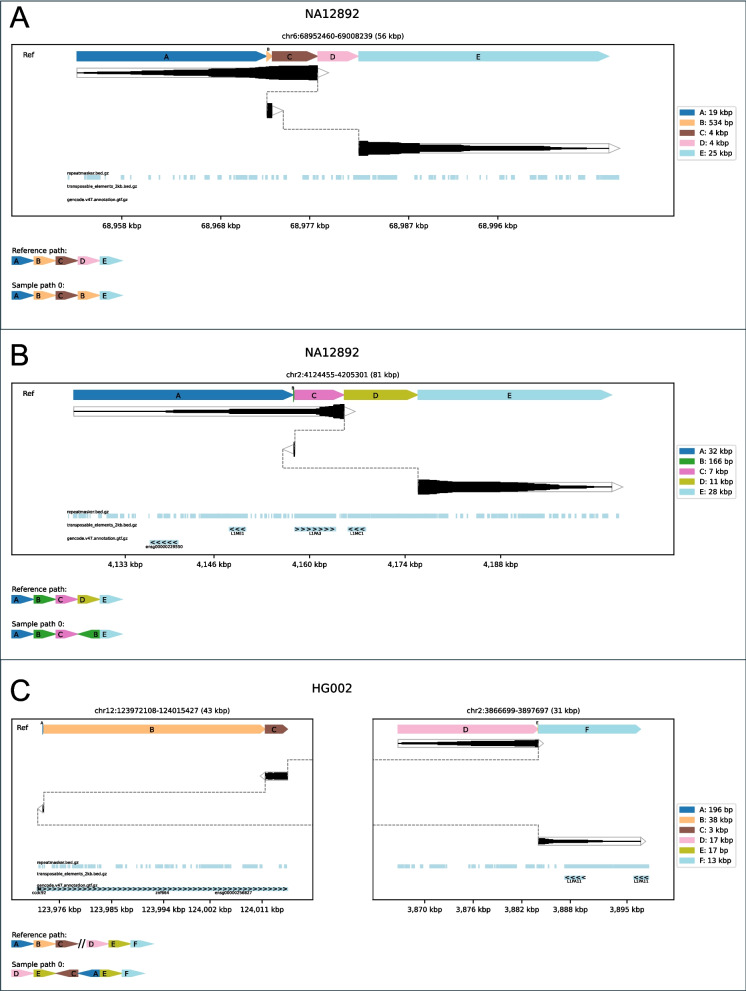
Examples of three complex SV categories: Plots of three complex SV types that are challenging to interpret without SVTopo representations. **A** Dup-del: non-tandem duplication followed by a deletion. **B** Nontandem-inv-del: An inverted non-tandem duplication followed by a deletion. **C** Balanced-trans-dup: A balanced translocation consisting of two blocks from chr12 translocated to chr2 and the duplication of a short region on chr2

Many of these SVs are challenging to visualize with existing tools but render clearly with SVTopo. This is demonstrated by examples of non-tandem duplications and balanced translocations with flanking duplication, as shown in Fig. 4. See also Supplementary Figs. 6–12 for more examples of these complex SV types with comparison to IGV and Ribbon visualizations, each containing notes to describe the category definition.

**Fig. 5:**
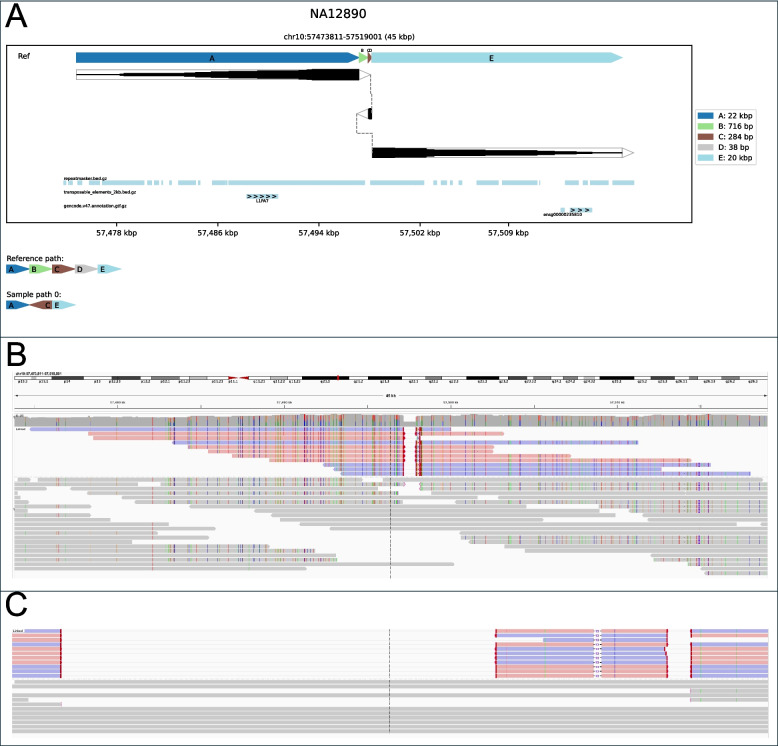
Example unbalanced inversion with flanking deletions: A 284 bp inversion flanked by a 716 bp deletion and a 38 bp deletion (category Del-inv-del). **A** SVTopo plot of region. **B** IGV screen capture of the region with linked supplementary alignments. **C** IGV image of the region, zoomed in to include only complex SV breakpoints

Inversions were the most common SV type plotted by SVTopo from these seven genomes (simple deletions and duplications being omitted by default) and were grouped into three categories: balanced (canonical) inversions (Inv, *n* = 44), with a genomic block reversed in orientation and directly reconnected; inversions with exactly one flanking deletion (Inv-del, n = 17); and inversions with flanking deletions on both sides (Inv-double-del, *n* = 25). While often not considered a complex SV (and omitted from complex SV categories in Fig. 2), balanced inversions are often quite challenging to visualize using standard techniques. Visualizations not designed for complex SVs struggle to show each of the component rearrangements in inversions distinctly, particularly for unbalanced inversions (See Fig. 5).

**Fig. 6:**
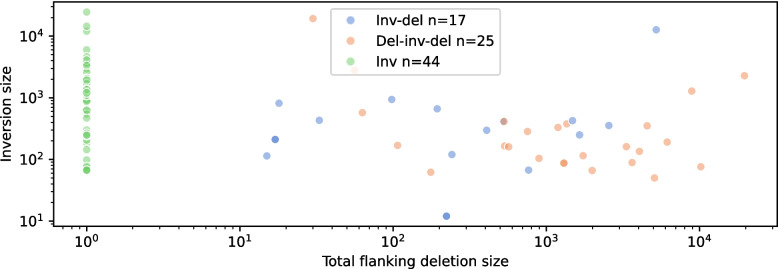
Balanced and unbalanced inversion sizes: Lengths of inverted blocks contrasted against flanking deletion sizes for inversions with 0–2 flanking deletions. Each axis is shown on log scale. Non-tandem inversions excluded

A total of 44 unique balanced inversion variants were plotted, 19 occurring in multiple samples to appear a total of 83 times. 17 distinct inversions with a single flanking deletion were plotted, appearing a total of 42 times; and 25 with two flanking deletions, appearing 52 times. Almost half (42 of 86) of the inversions were accompanied by flanking deletions (see Fig. 6 and Supplementary Table 2).

### SVTopo table viewer

**Fig. 7:**
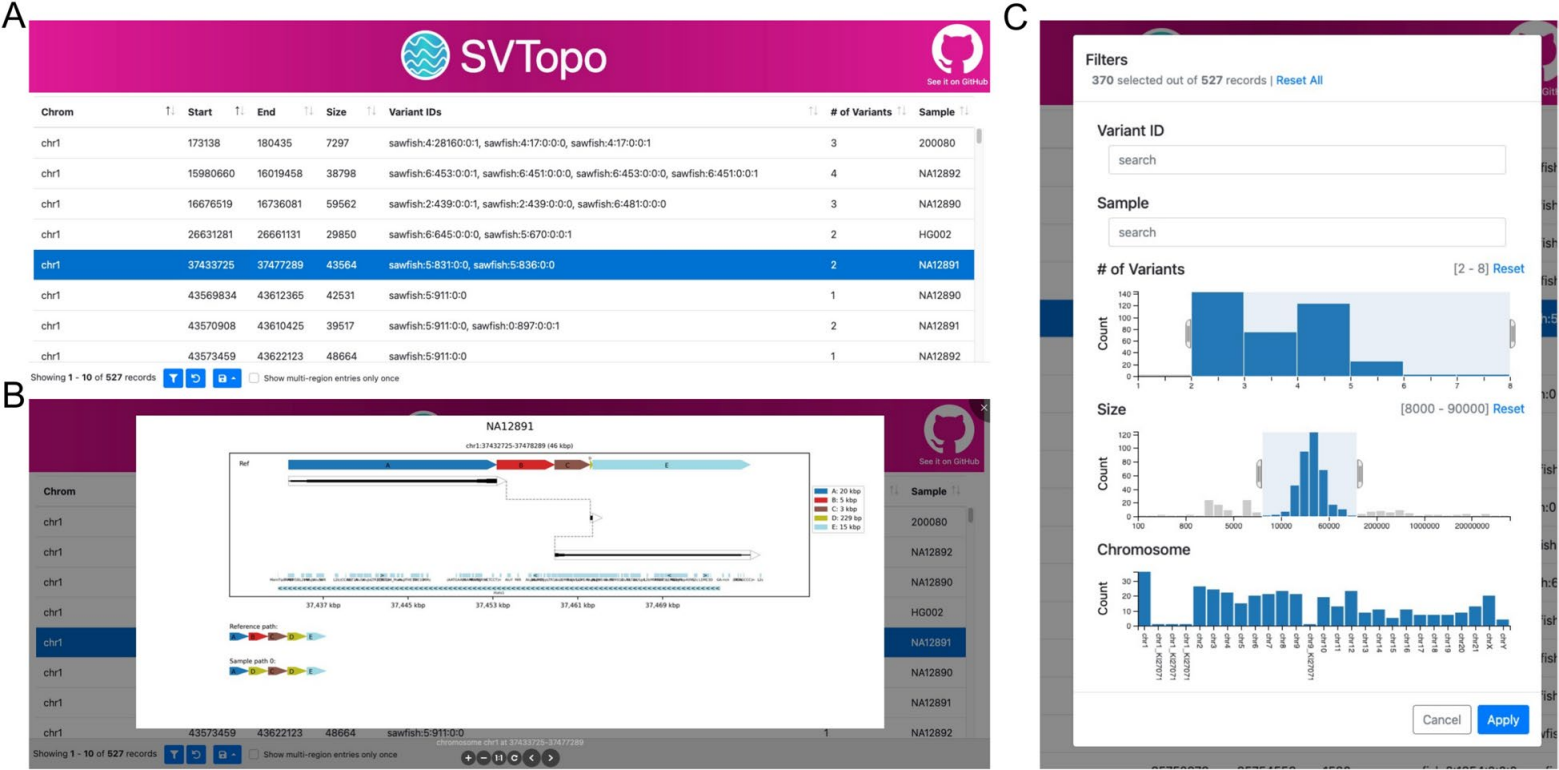
SVTopo table viewer. **A** A screen capture of the table viewer, showing included table columns. **B** A screen capture of a complex SV image displayed by clicking a table row. Image shows a deletion and non-tandem duplication. **C** Viewer filtering window, with filtering fields for variant ID, sample ID, number of variants, region size, and chromosome. Filters shown select for 2–8 variants per image, a size range of 8,000–90,000 bp, and all chromosomes

In addition to providing simple visualizations for SV review, SVTopo automatically creates a feature-rich serverless HTML-based viewing table to organize and present images (see Fig. 7). This viewer is a self-contained web page, with built-in filtering tools to allow users to rapidly identify the complex SVs most relevant to a research priority (such as reviewing images relevant to a region or sample of interest). The viewer can be deployed locally for a single user or remotely if multi-user access is desired (example viewer publicly available [30]). The viewer main page consists of a variant region table, with a row for each genomic window represented. Clicking a row displays the image. Columns in the table show the chromosome, start, end, region size, associated variant IDs (if available), number of variant IDs represented in the image (if available), and sample ID. The table allows a user to filter by clicking a variant ID, number of variants field, or sample ID. A filtering options window also allows filtering on region size ranges and target chromosomes.

## Discussion

Complex SVs (networks of two or more connected genomic breaks in a sample haplotype, excluding balanced inversions) are difficult to visualize using standard genome browsing tools. SVTopo provides a new toolkit for visual analysis of germline complex SVs, with an easy-to-understand plot layout and simple deployment interface. Analysis of seven unrelated genomes revealed 101 unique complex SVs in ten categories. SVTopo will be especially useful for 1) understanding the complexity of SV calls in whole genome analysis, with total per-genome complex SV counts that remain tractable for human review, and 2) reviewing genome structure in specific regions to better understand the functional impact of complex SVs. SVTopo can be used as a standalone visualization tool or in tandem with existing tools such as IGV, Ribbon, etc., enabling multiple levels of visual review for complex SVs.

The SVTopo images improve complex SV interpretation, conveying information with individual blocks of genomic material. Explicit block orientations and connections between blocks inform the user of how genomic regions are arranged, information that is essential for understanding potential impacts on genetic disease from novel adjacencies and lost or duplicated material. Examples of easily interpretable SVTopo representations of complex rearrangements in Supplementary Figs. 6–12 show these advantages compared to current methods (IGV and Ribbon). In balanced translocations, for example (Supplementary Fig. 6), existing tools may mask small flanking duplications or make it difficult to identify and view donor sites. Similarly, the many varieties of possible non-tandem inversions and duplications are challenging to understand with existing tools (see Supplementary Figs. 8–12), as showing such rearrangements with a focus on either reference or sample ordering of blocks can mask the relationships between the two. An inversion between two deletions (Fig. 5) can be difficult to differentiate from a pair of deletions around a non-inverted region (Supplementary Fig. 7).

The sample frequency of some complex SVs, appearing in multiple unrelated genomes even within this small cohort, indicates likely high population frequencies. Inversions, often considered a simple SV type, are shown here to occur nearly as often with flanking deletions as without (44 unique balanced inversions vs 42 with flanking deletions). This finding also demonstrates how better variant visualization can improve our understanding of the effects of inversion on genomes and the mechanisms that drive structural rearrangement. The 101 complex SVs analyzed in these genomes and their variety across ten distinct categories show that it is not uncommon for a genome to contain multiple distinct types of complex rearrangements.

The SVTopo table viewer, designed to enhance genome analysis for small and large projects or teams, is a significant part of the SVTopo toolkit. The ease of deployment and filtering options provides necessary assistance to researchers for identification of complex SVs relevant to important genomic questions and improve the utility of SVTopo for sharing results among teams or collaborators.

## Conclusions

SVTopo makes complex structural rearrangements easier to understand and interpret. It increases the utility of high accuracy long reads to more fully demonstrate the complexity of the genome, recovering insights into highly complex genomic rearrangement that otherwise escape notice or human comprehension. SVTopo performs reconstructions necessary to reveal the structure of complex rearrangements in simple and human-accessible ways.

## Supplementary Information

Supplementary Material 1.

Supplementary Material 2.

Supplementary Material 3.

## Data Availability

All data and software are publicly available at [https://zenodo.org/records/15086376](https:/zenodo.org/records/15086376).
