# Supplementary material for: Complex structural variant visualization with SVTopo
Source: BMC Genomics. 2025 Oct 9;26:903. doi: 10.1186/s12864-025-12088-6 (PMC12512824; doi:10.1186/s12864-025-12088-6)
Supplement: Supplementary file 1 — Supplementary Material 1. [file 12864_2025_12088_MOESM1_ESM.pptx]

## Slide 1
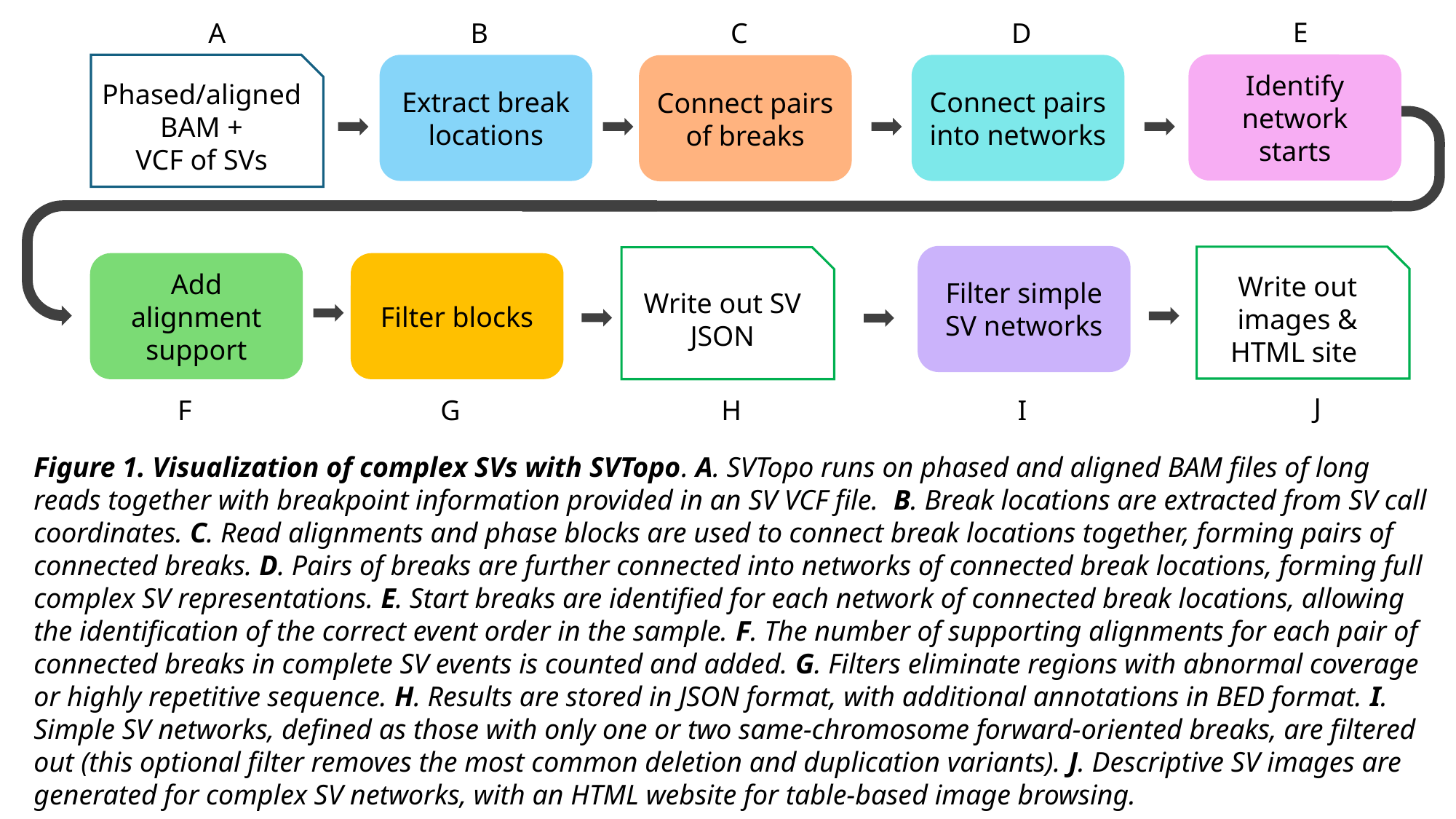

E
A
B
C
D
Identify network starts
Phased/aligned BAM +
VCF of SVs
Extract break locations
Connect pairs into networks
Connect pairs of breaks
Filter simple SV networks
Write out images & HTML site
Write out SV JSON
Add alignment support
Filter blocks
J
F
G
H
I
Figure 1. Visualization of complex SVs with SVTopo. A. SVTopo runs on phased and aligned BAM files of long reads together with breakpoint information provided in an SV VCF file.  B. Break locations are extracted from SV call coordinates. C. Read alignments and phase blocks are used to connect break locations together, forming pairs of connected breaks. D. Pairs of breaks are further connected into networks of connected break locations, forming full complex SV representations. E. Start breaks are identified for each network of connected break locations, allowing the identification of the correct event order in the sample. F. The number of supporting alignments for each pair of connected breaks in complete SV events is counted and added. G. Filters eliminate regions with abnormal coverage or highly repetitive sequence. H. Results are stored in JSON format, with additional annotations in BED format. I. Simple SV networks, defined as those with only one or two same-chromosome forward-oriented breaks, are filtered out (this optional filter removes the most common deletion and duplication variants). J. Descriptive SV images are generated for complex SV networks, with an HTML website for table-based image browsing.

## Slide 2
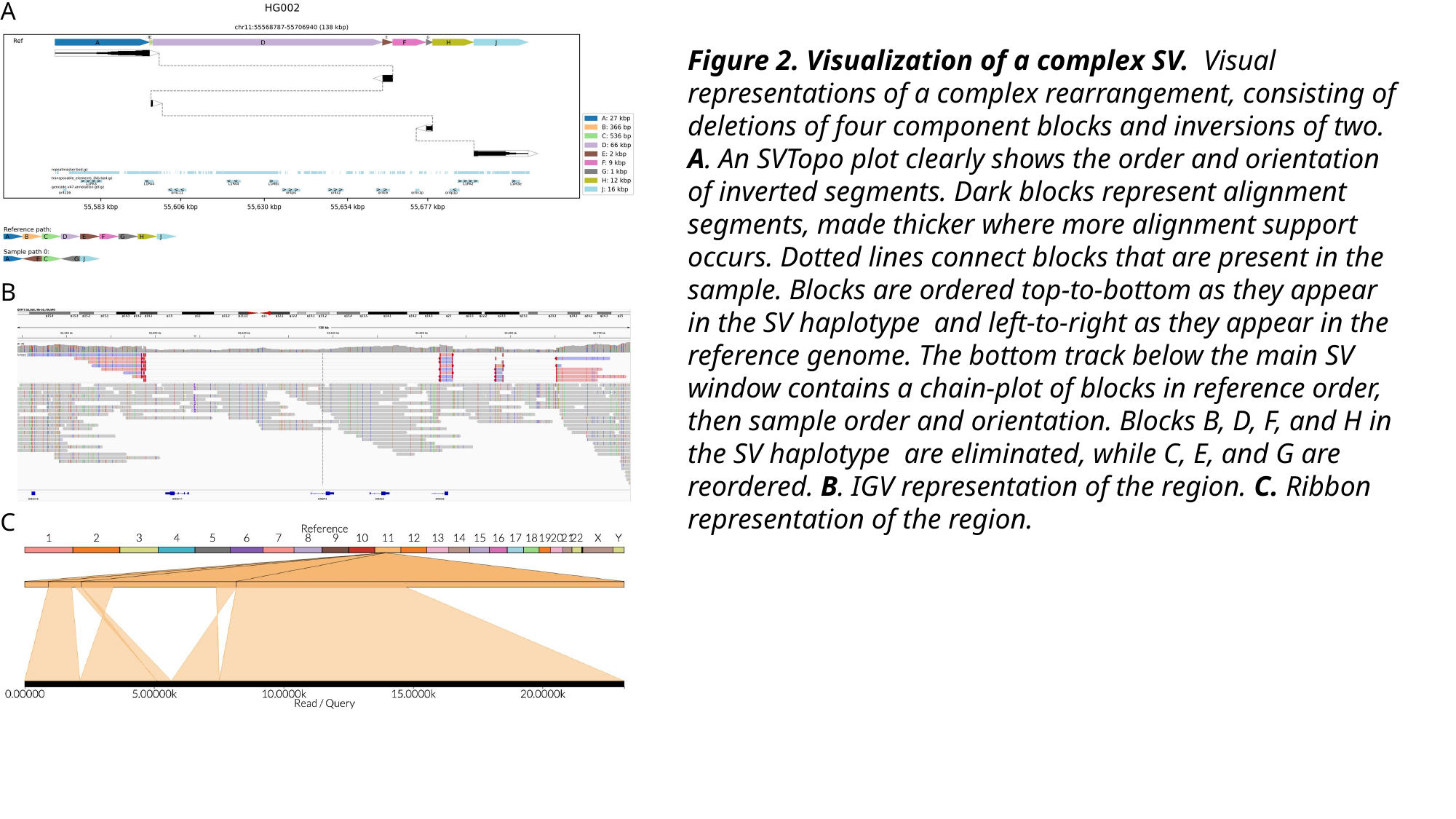

A
Figure 2. Visualization of a complex SV.  Visual representations of a complex rearrangement, consisting of deletions of four component blocks and inversions of two. A. An SVTopo plot clearly shows the order and orientation of inverted segments. Dark blocks represent alignment segments, made thicker where more alignment support occurs. Dotted lines connect blocks that are present in the sample. Blocks are ordered top-to-bottom as they appear in the SV haplotype  and left-to-right as they appear in the reference genome. The bottom track below the main SV window contains a chain-plot of blocks in reference order, then sample order and orientation. Blocks B, D, F, and H in the SV haplotype  are eliminated, while C, E, and G are reordered. B. IGV representation of the region. C. Ribbon representation of the region.
B
C

## Slide 3
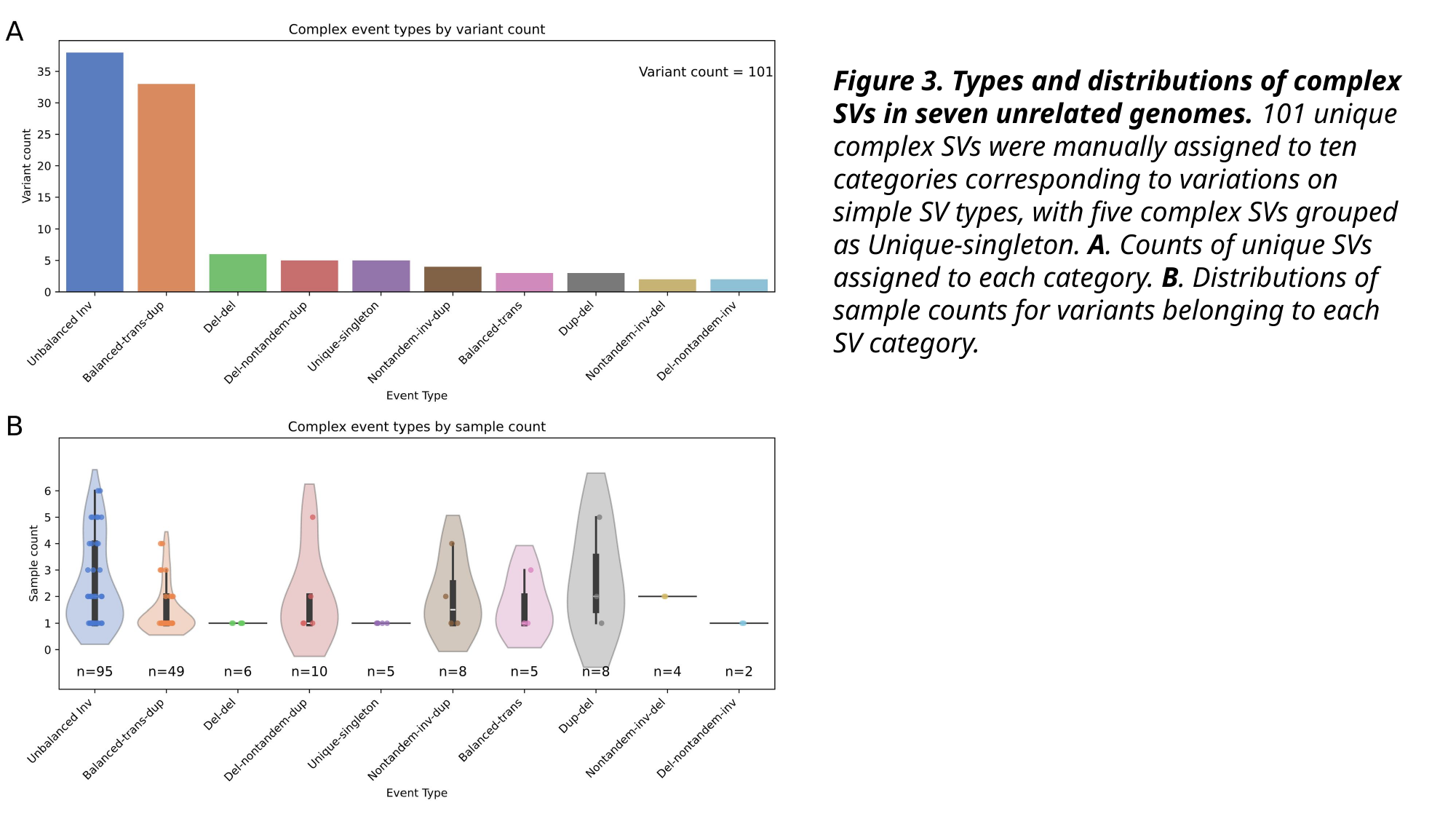

Figure 3. Types and distributions of complex SVs in seven unrelated genomes. 101 unique complex SVs were manually assigned to ten categories corresponding to variations on simple SV types, with five complex SVs grouped as Unique-singleton. A. Counts of unique SVs assigned to each category. B. Distributions of sample counts for variants belonging to each SV category.

## Slide 4
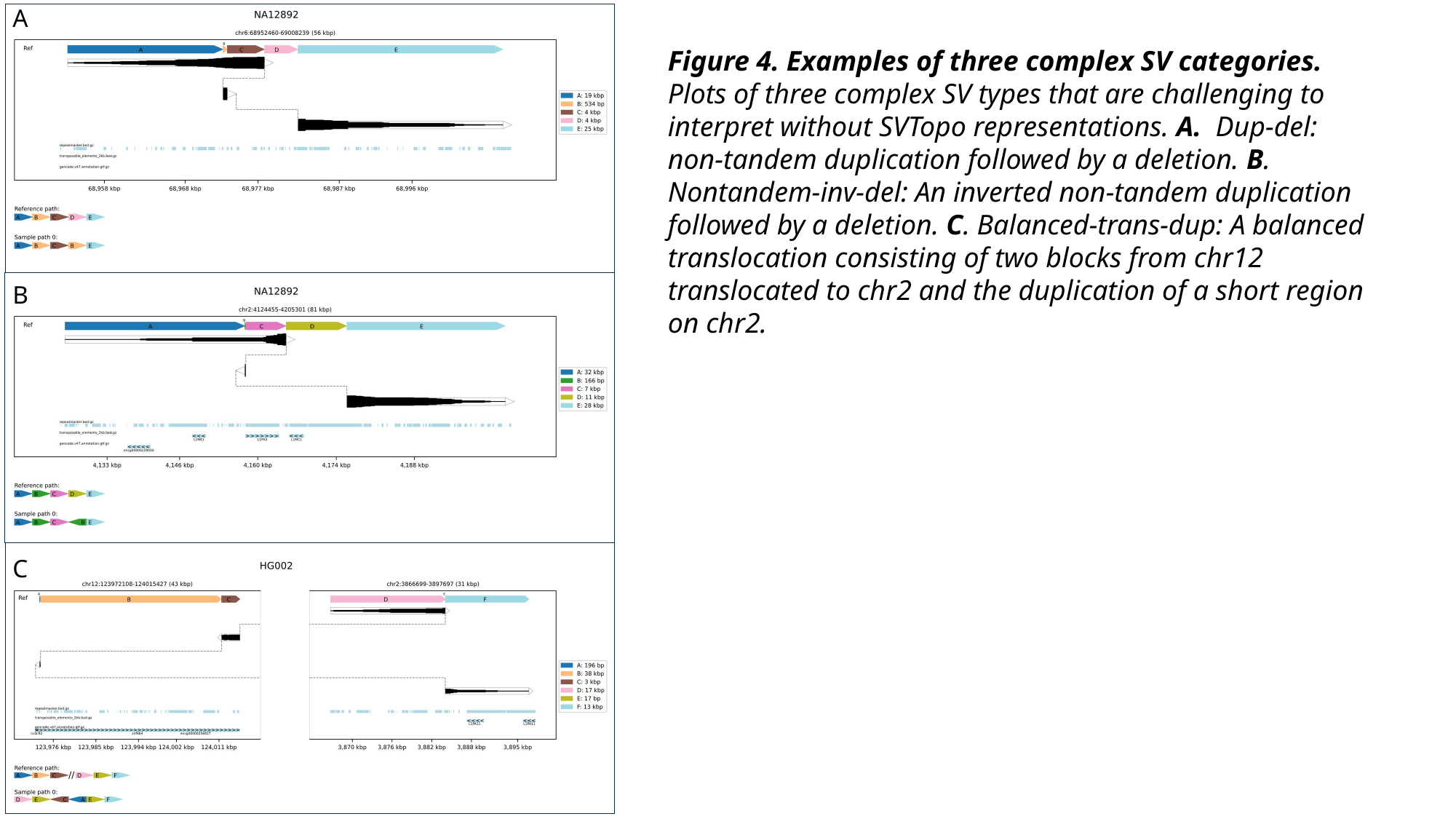

A
Figure 4. Examples of three complex SV categories. Plots of three complex SV types that are challenging to interpret without SVTopo representations. A. Dup-del: non-tandem duplication followed by a deletion. B. Nontandem-inv-del: An inverted non-tandem duplication followed by a deletion. C. Balanced-trans-dup: A balanced translocation consisting of two blocks from chr12 translocated to chr2 and the duplication of a short region on chr2.
B
C

## Slide 5
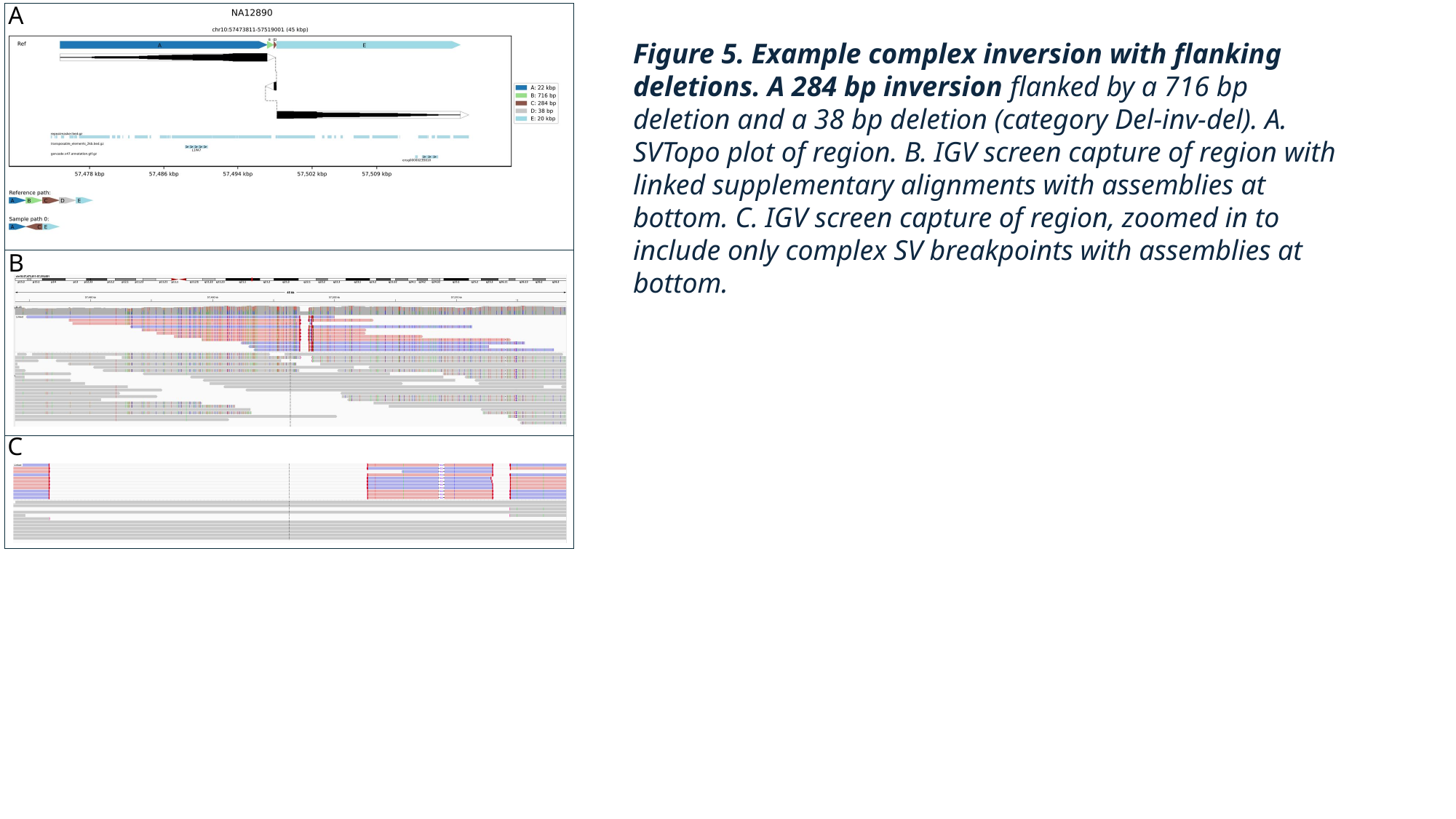

A
Figure 5. Example complex inversion with flanking deletions. A 284 bp inversion flanked by a 716 bp deletion and a 38 bp deletion (category Del-inv-del). A. SVTopo plot of region. B. IGV screen capture of region with linked supplementary alignments with assemblies at bottom. C. IGV screen capture of region, zoomed in to include only complex SV breakpoints with assemblies at bottom.
B
C

## Slide 6
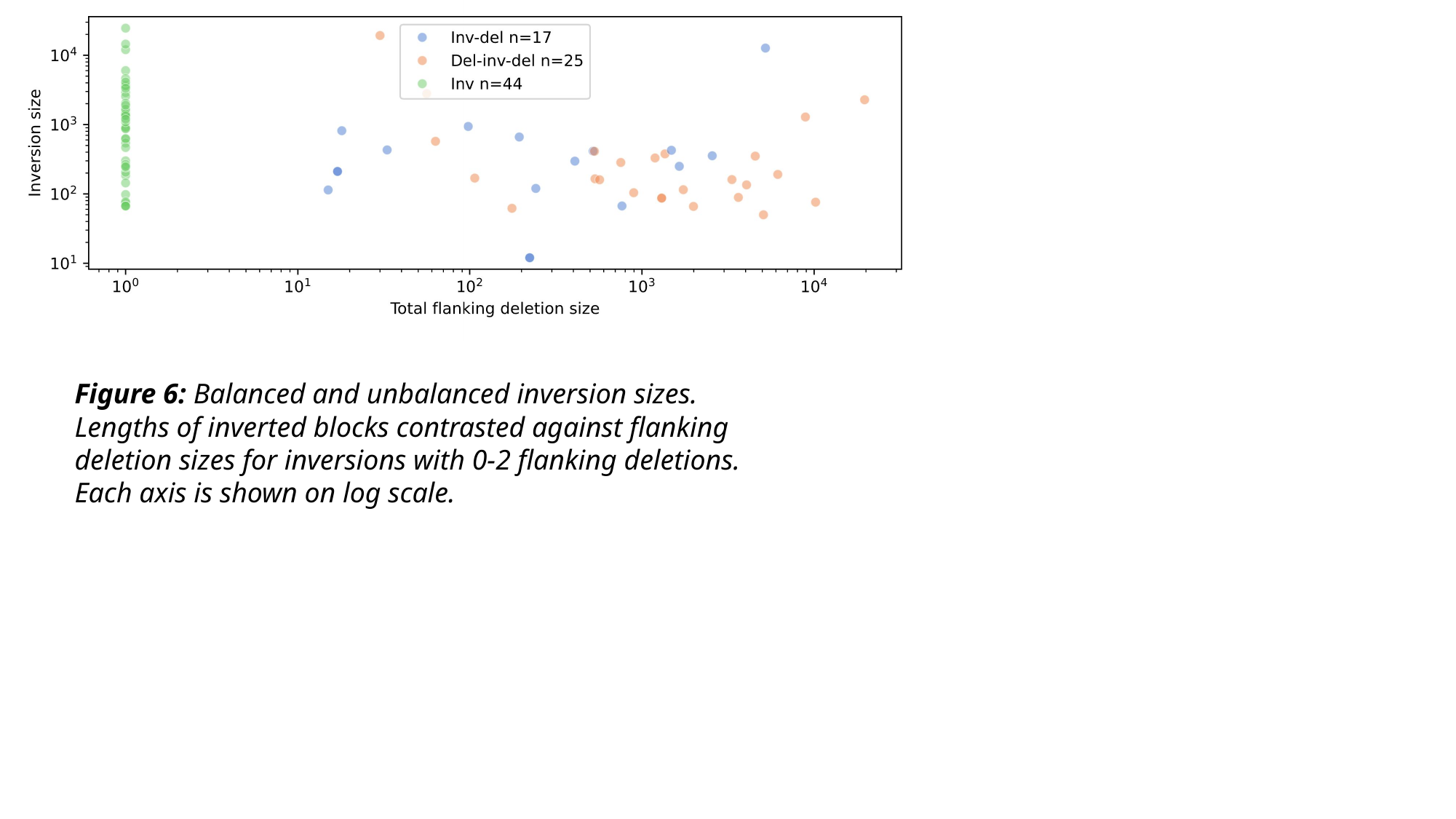

Figure 6: Balanced and unbalanced inversion sizes. Lengths of inverted blocks contrasted against flanking deletion sizes for inversions with 0-2 flanking deletions. Each axis is shown on log scale.

## Slide 7
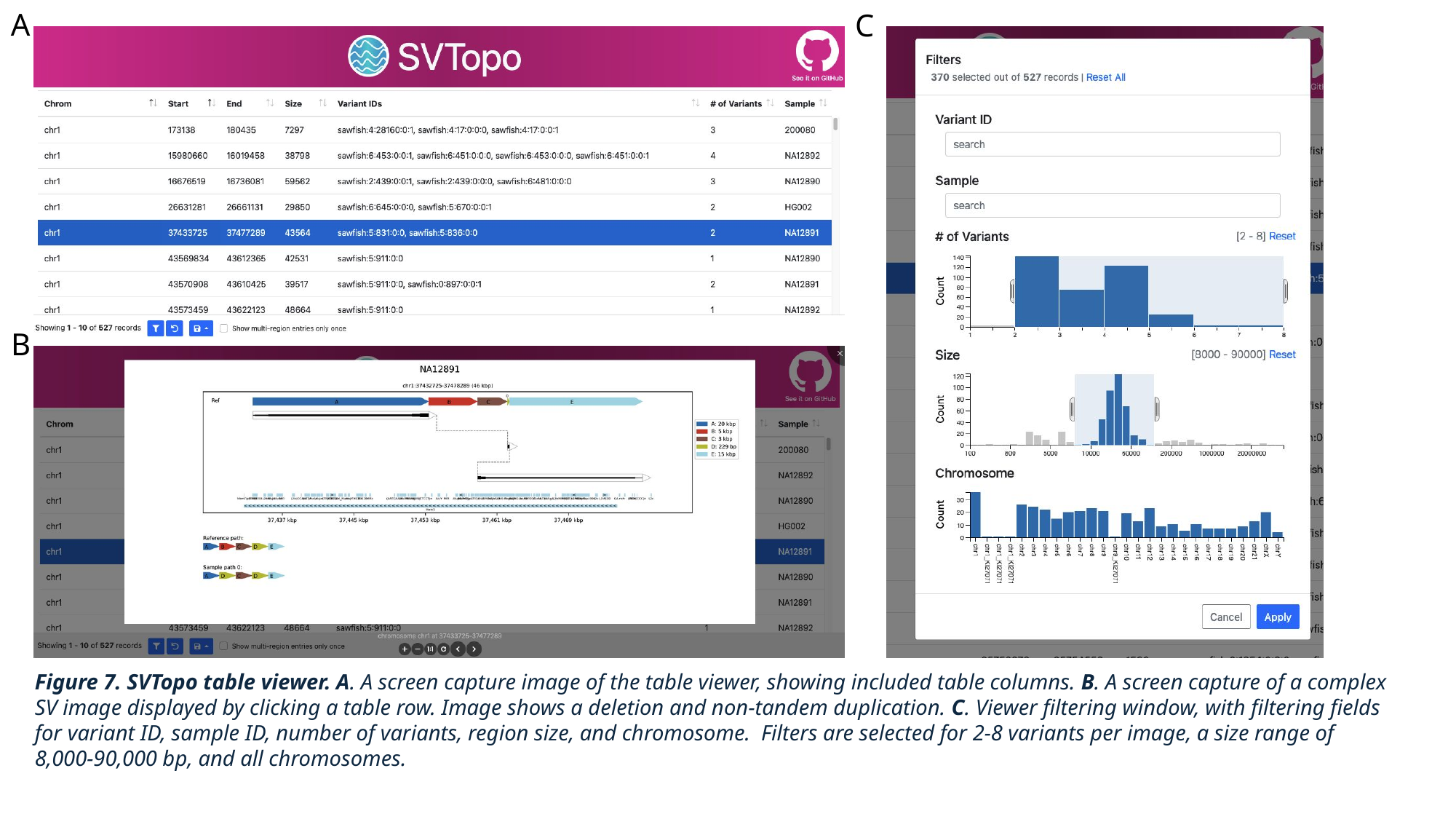

A
C
B
Figure 7. SVTopo table viewer. A. A screen capture image of the table viewer, showing included table columns. B. A screen capture of a complex SV image displayed by clicking a table row. Image shows a deletion and non-tandem duplication. C. Viewer filtering window, with filtering fields for variant ID, sample ID, number of variants, region size, and chromosome. Filters are selected for 2-8 variants per image, a size range of 8,000-90,000 bp, and all chromosomes.

## Slide 8
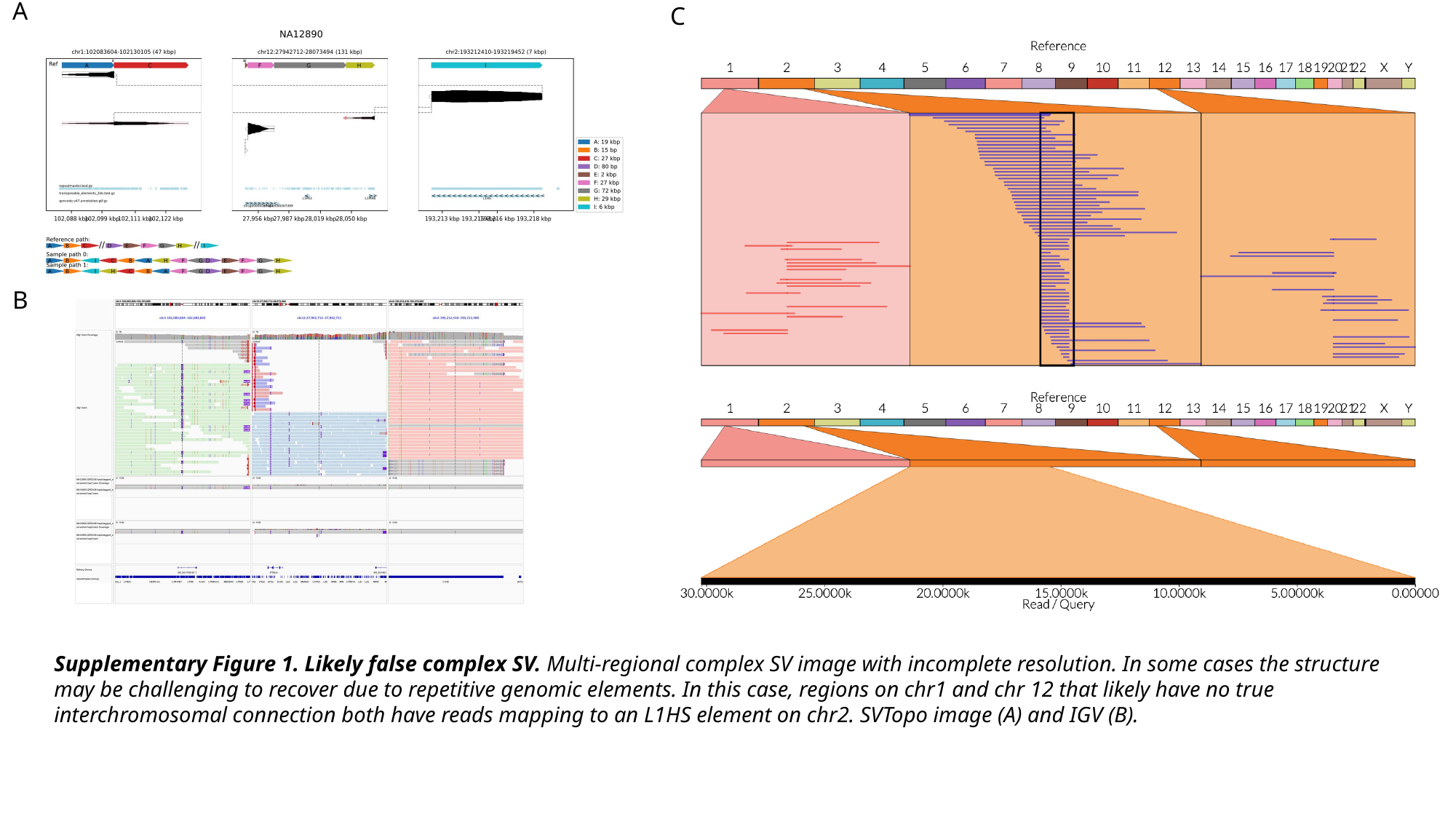

A
C
B
Supplementary Figure 1. Likely false complex SV. Multi-regional complex SV image with incomplete resolution. In some cases the structure may be challenging to recover due to repetitive genomic elements. In this case, regions on chr1 and chr 12 that likely have no true interchromosomal connection both have reads mapping to an L1HS element on chr2. SVTopo image (A) and IGV (B).

## Slide 9
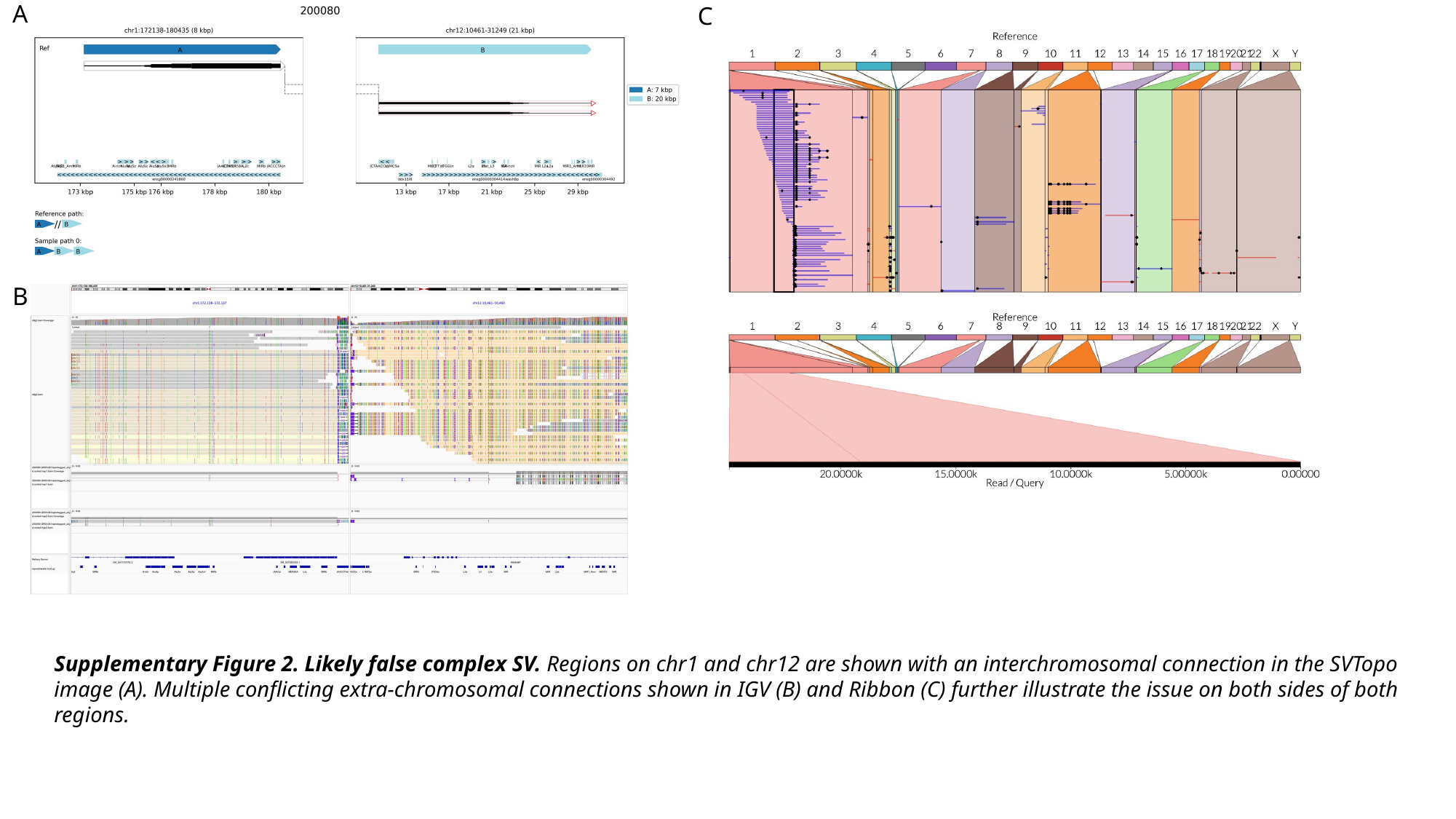

A
C
B
Supplementary Figure 2. Likely false complex SV. Regions on chr1 and chr12 are shown with an interchromosomal connection in the SVTopo image (A). Multiple conflicting extra-chromosomal connections shown in IGV (B) and Ribbon (C) further illustrate the issue on both sides of both regions.

## Slide 10
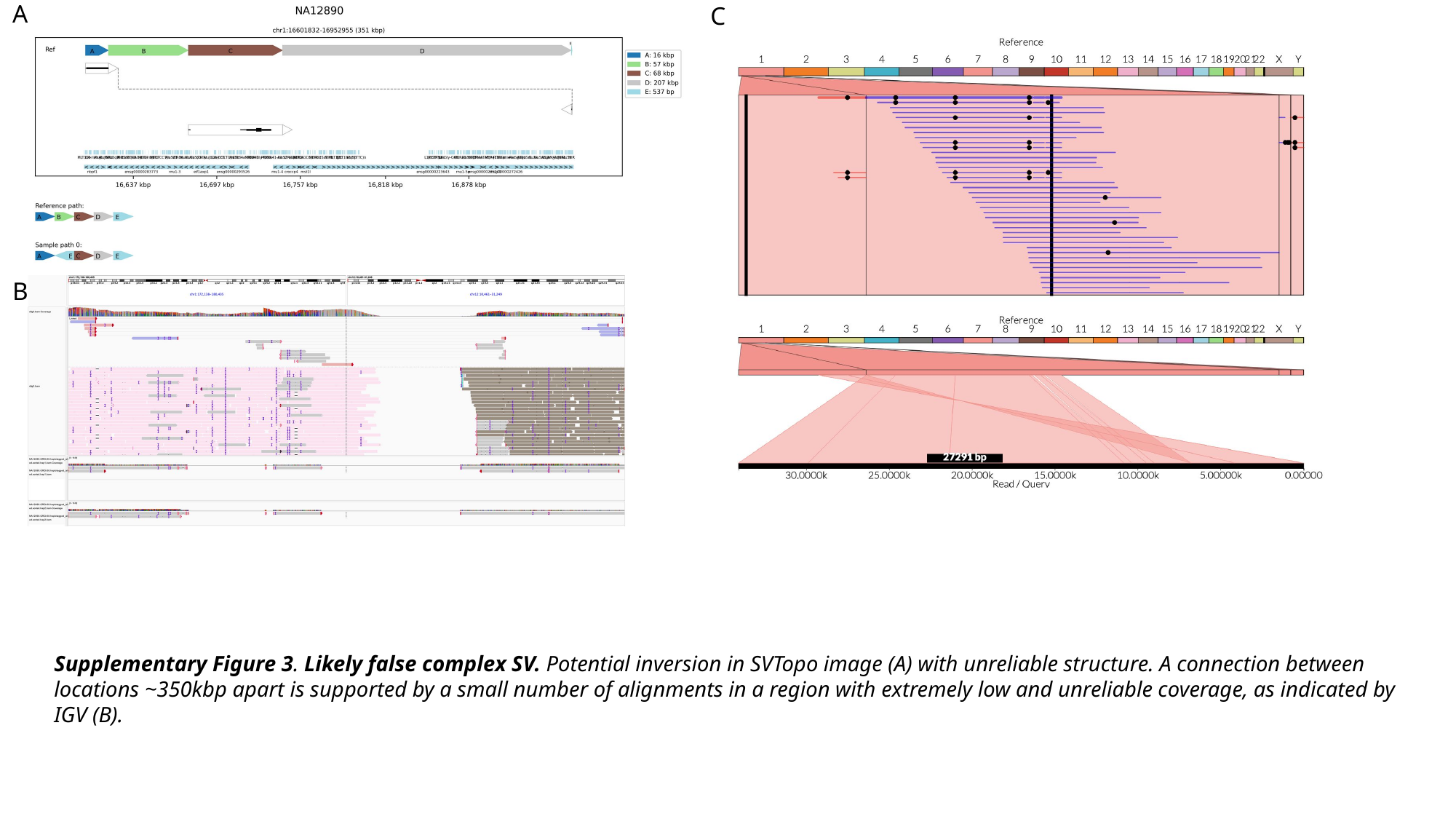

A
C
B
Supplementary Figure 3. Likely false complex SV. Potential inversion in SVTopo image (A) with unreliable structure. A connection between locations ~350kbp apart is supported by a small number of alignments in a region with extremely low and unreliable coverage, as indicated by IGV (B).

## Slide 11
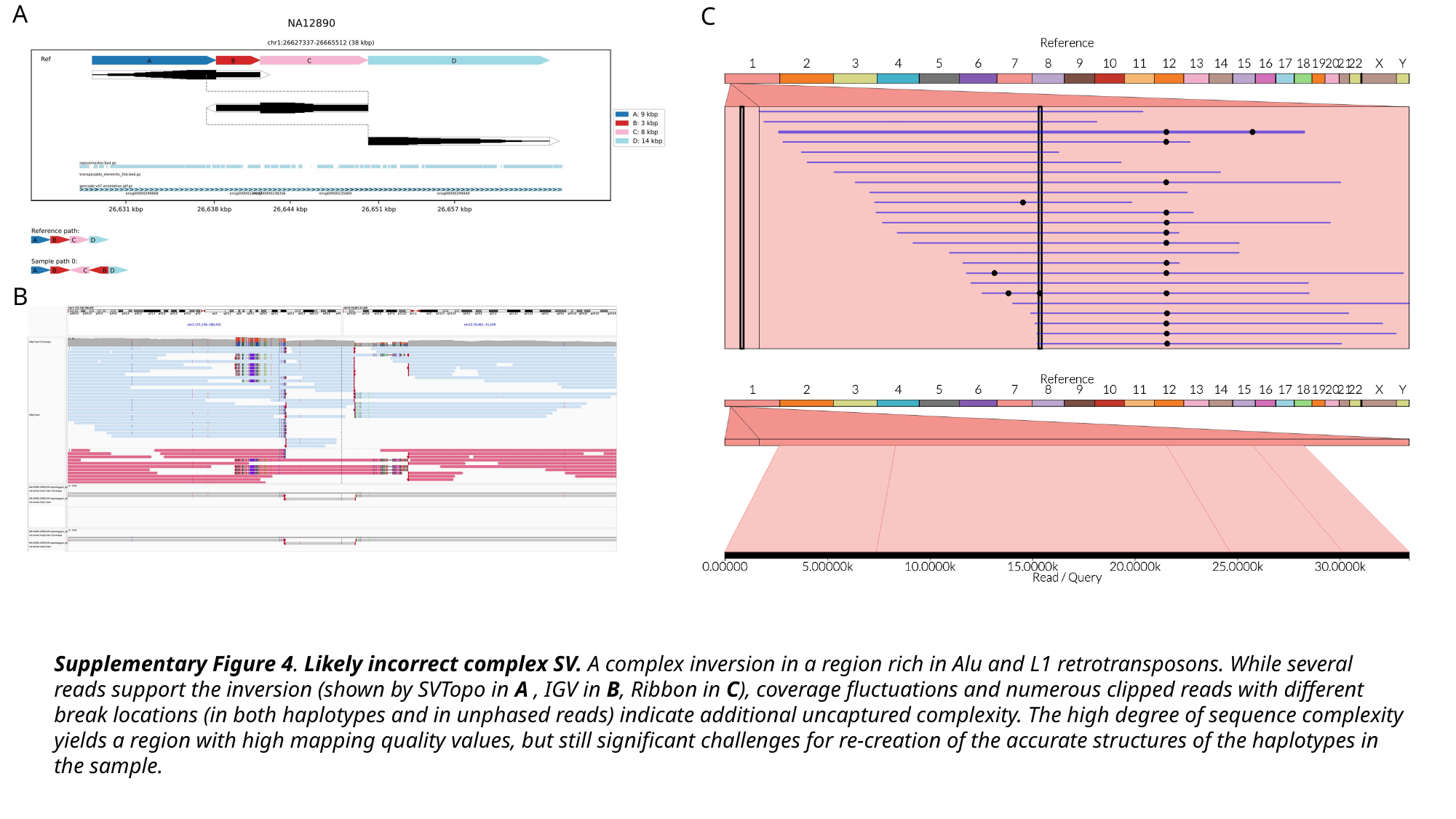

A
C
B
Supplementary Figure 4. Likely incorrect complex SV. A complex inversion in a region rich in Alu and L1 retrotransposons. While several reads support the inversion (shown by SVTopo in A , IGV in B, Ribbon in C), coverage fluctuations and numerous clipped reads with different break locations (in both haplotypes and in unphased reads) indicate additional uncaptured complexity. The high degree of sequence complexity yields a region with high mapping quality values, but still significant challenges for re-creation of the accurate structures of the haplotypes in the sample.

## Slide 12
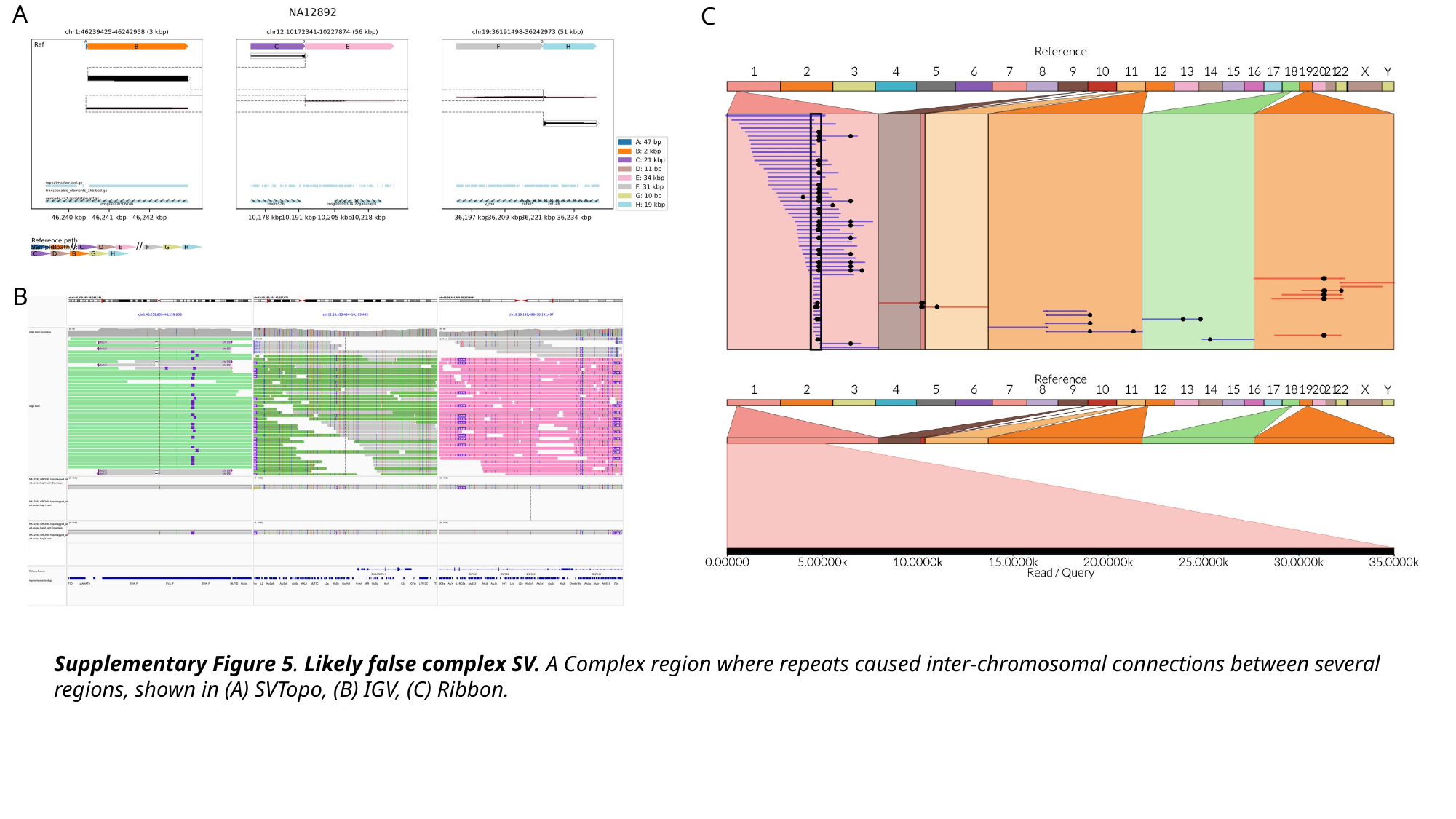

A
C
B
Supplementary Figure 5. Likely false complex SV. A Complex region where repeats caused inter-chromosomal connections between several regions, shown in (A) SVTopo, (B) IGV, (C) Ribbon.

## Slide 13
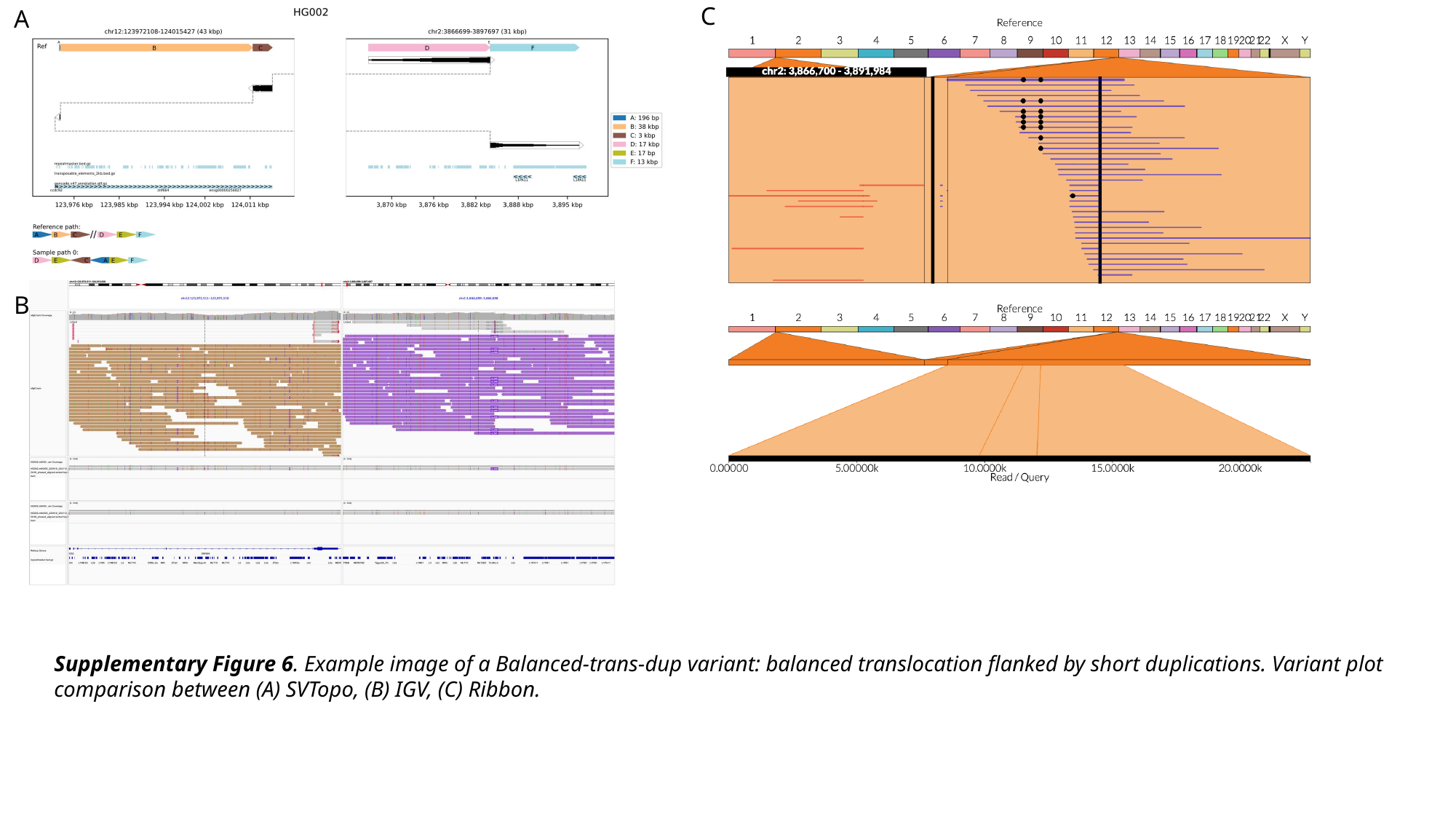

C
A
B
Supplementary Figure 6. Example image of a Balanced-trans-dup variant: balanced translocation flanked by short duplications. Variant plot comparison between (A) SVTopo, (B) IGV, (C) Ribbon.

## Slide 14
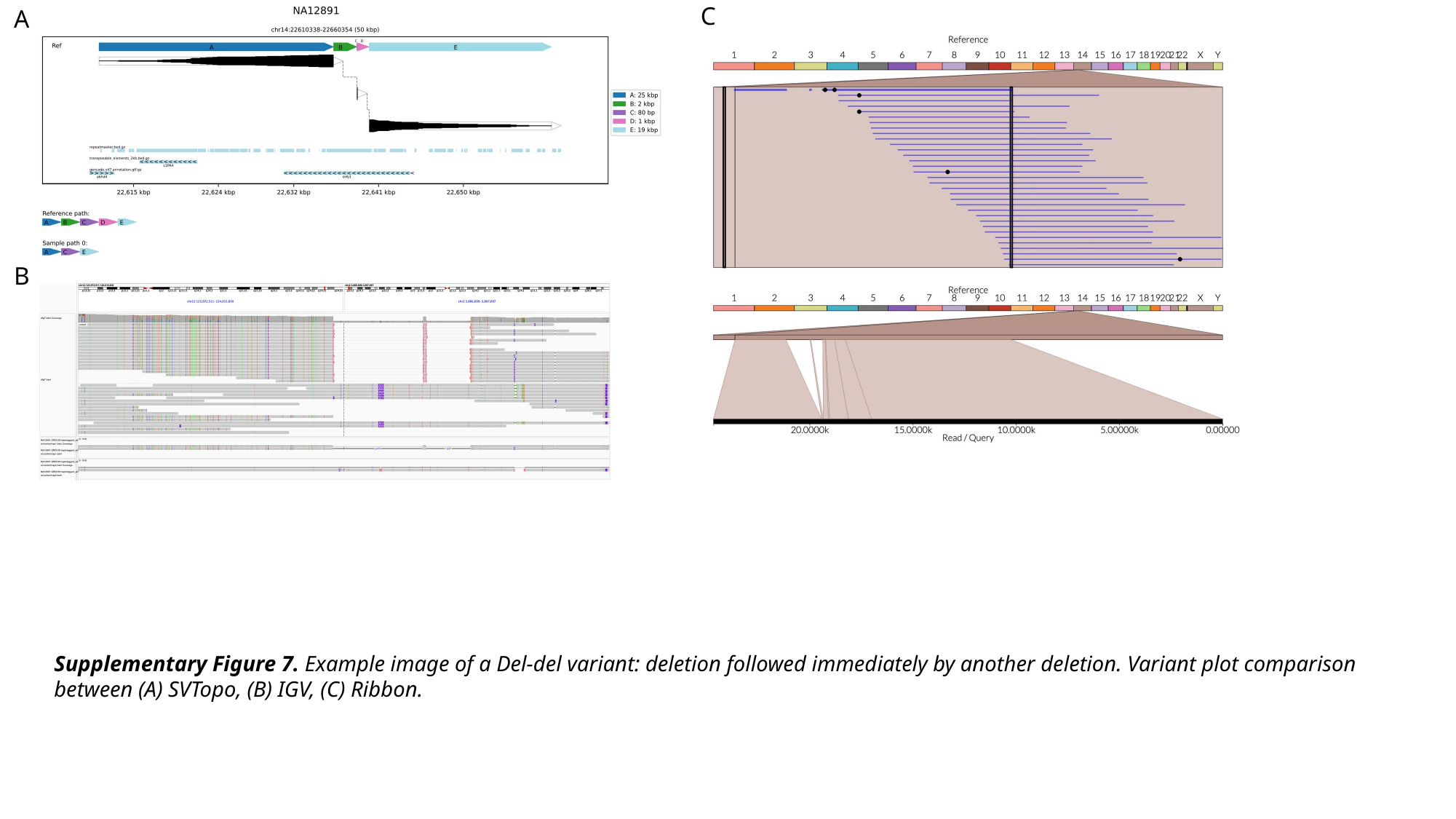

C
A
B
Supplementary Figure 7. Example image of a Del-del variant: deletion followed immediately by another deletion. Variant plot comparison between (A) SVTopo, (B) IGV, (C) Ribbon.

## Slide 15
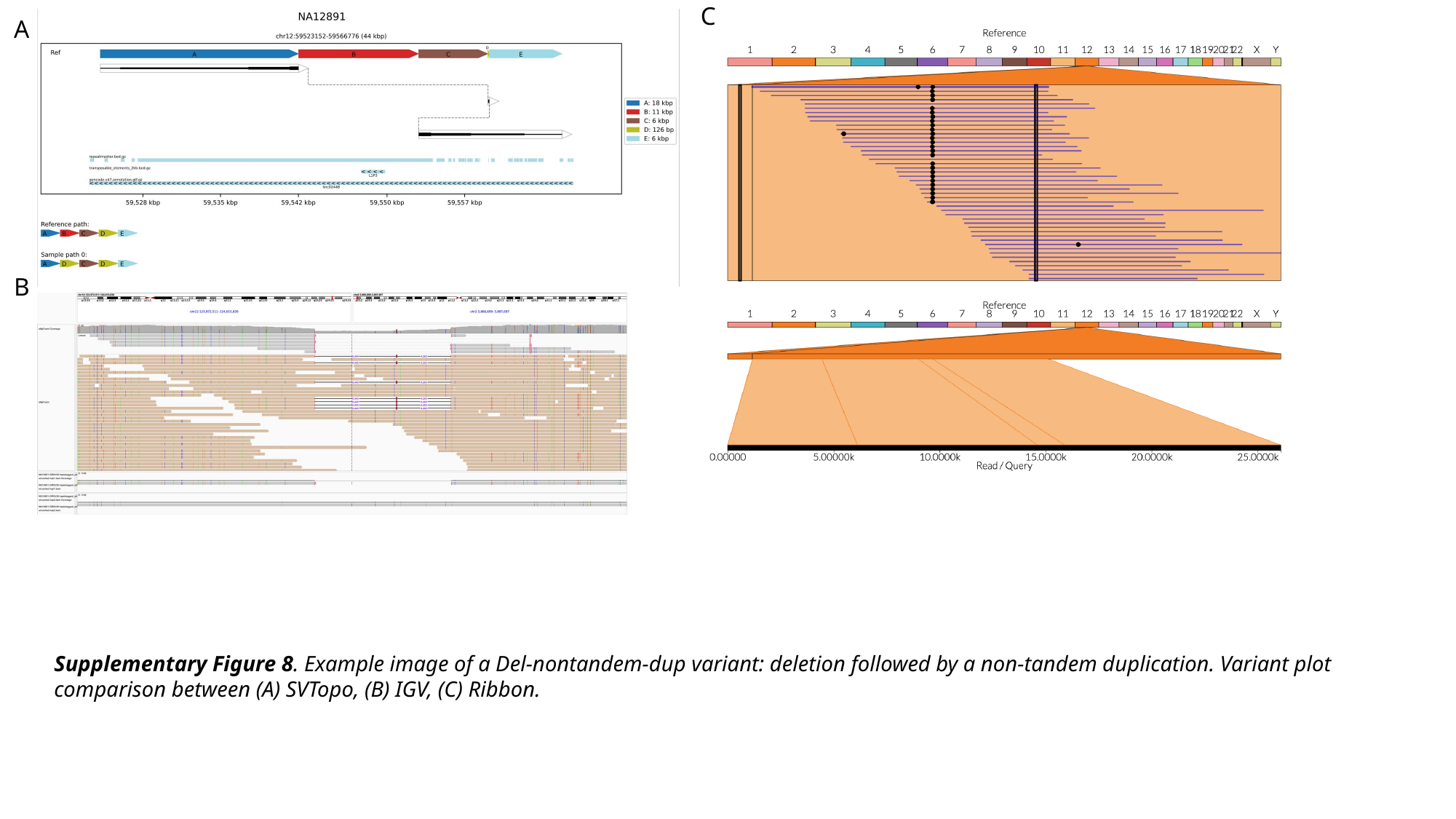

C
A
B
Supplementary Figure 8. Example image of a Del-nontandem-dup variant: deletion followed by a non-tandem duplication. Variant plot comparison between (A) SVTopo, (B) IGV, (C) Ribbon.

## Slide 16
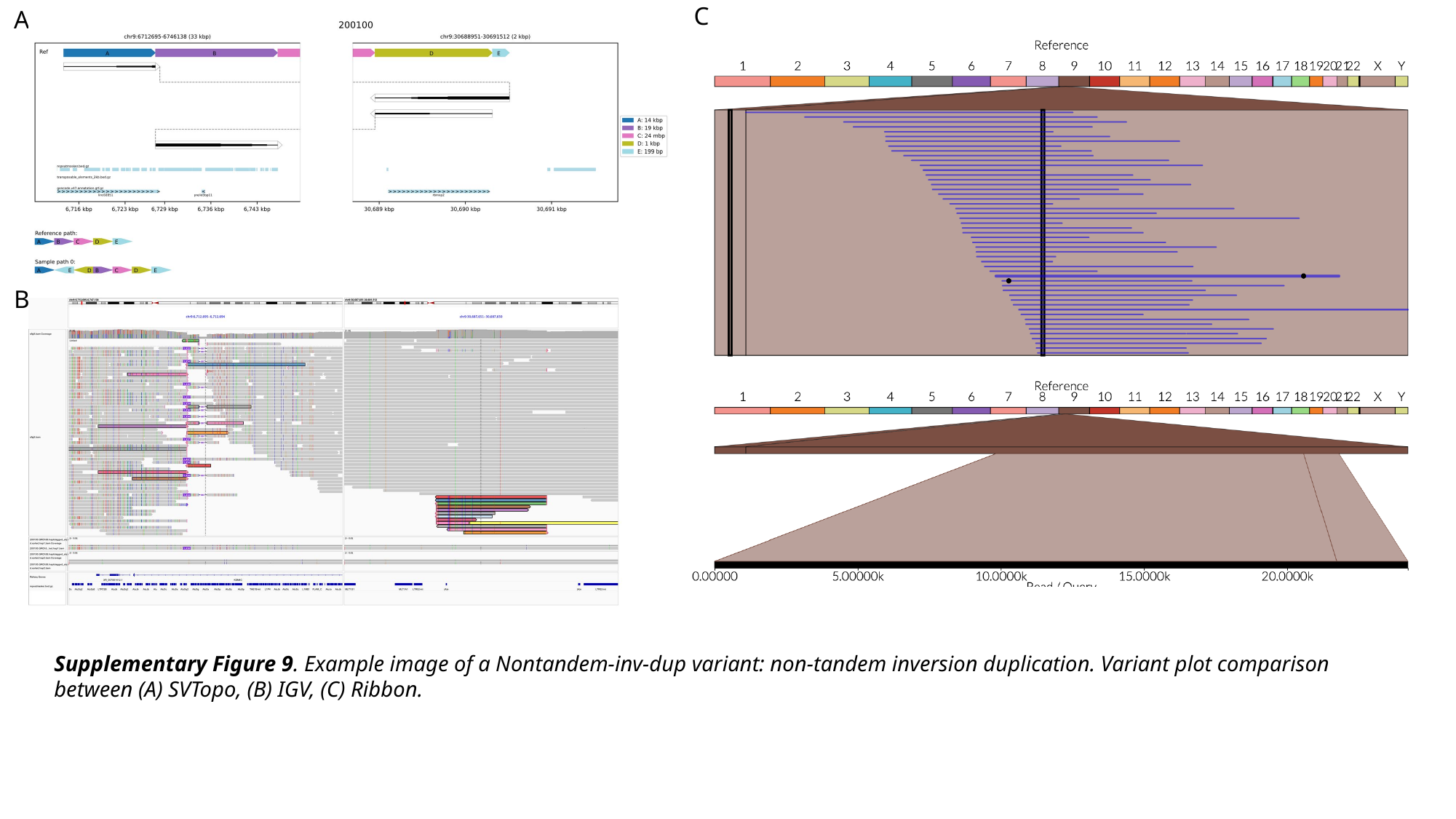

C
A
B
Supplementary Figure 9. Example image of a Nontandem-inv-dup variant: non-tandem inversion duplication. Variant plot comparison between (A) SVTopo, (B) IGV, (C) Ribbon.

## Slide 17
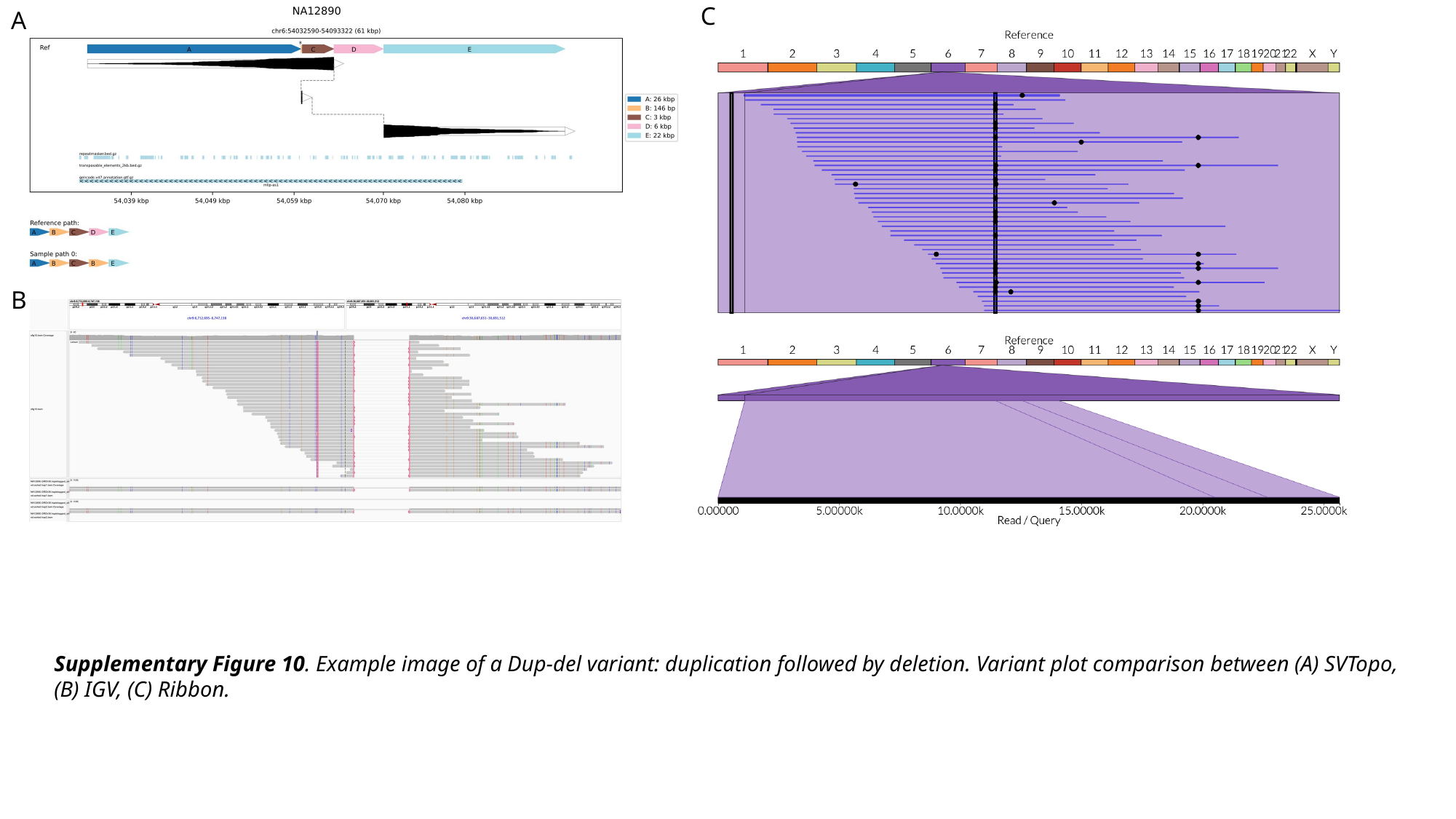

C
A
B
Supplementary Figure 10. Example image of a Dup-del variant: duplication followed by deletion. Variant plot comparison between (A) SVTopo, (B) IGV, (C) Ribbon.

## Slide 18
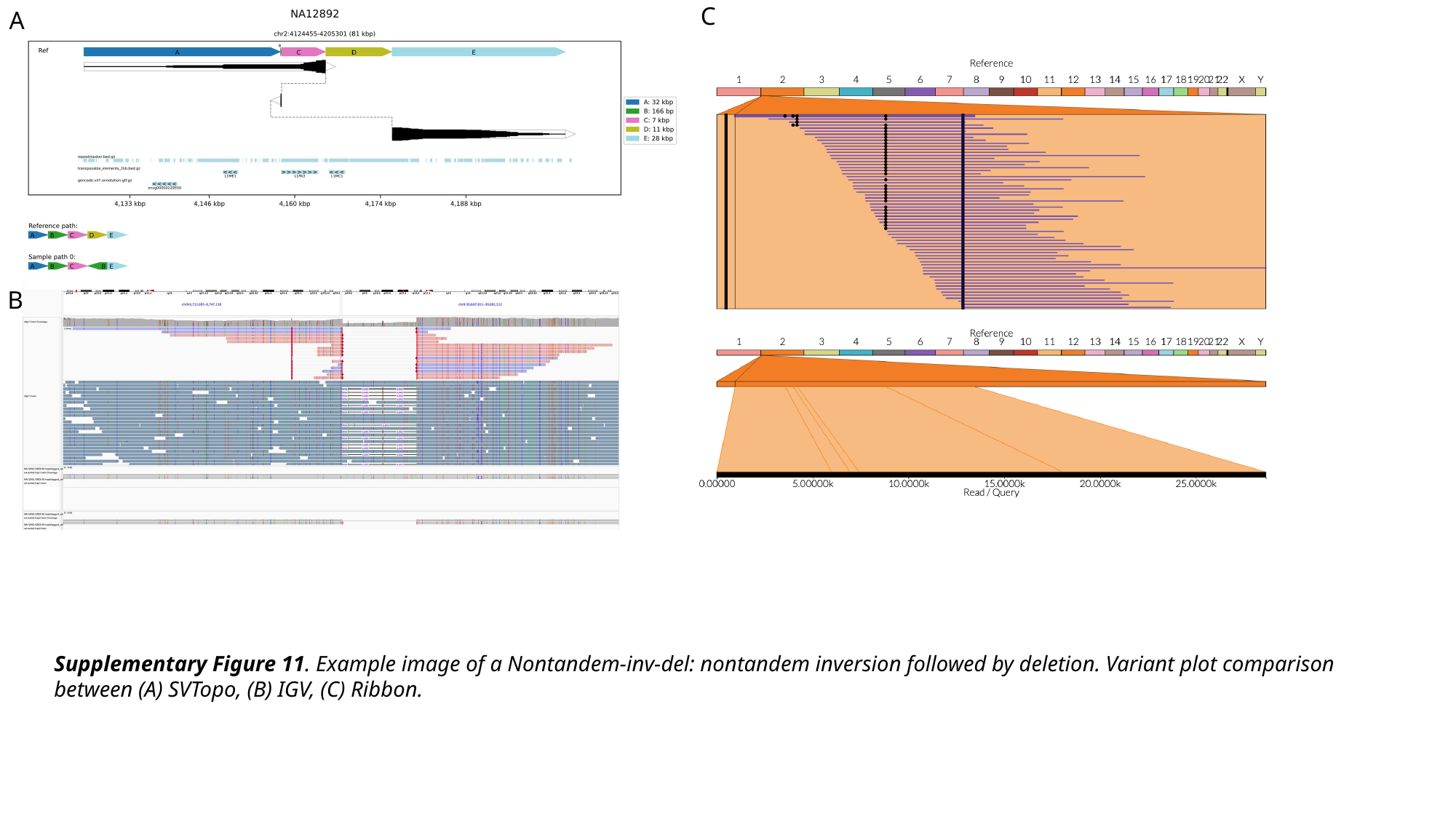

C
A
B
Supplementary Figure 11. Example image of a Nontandem-inv-del: nontandem inversion followed by deletion. Variant plot comparison between (A) SVTopo, (B) IGV, (C) Ribbon.

## Slide 19
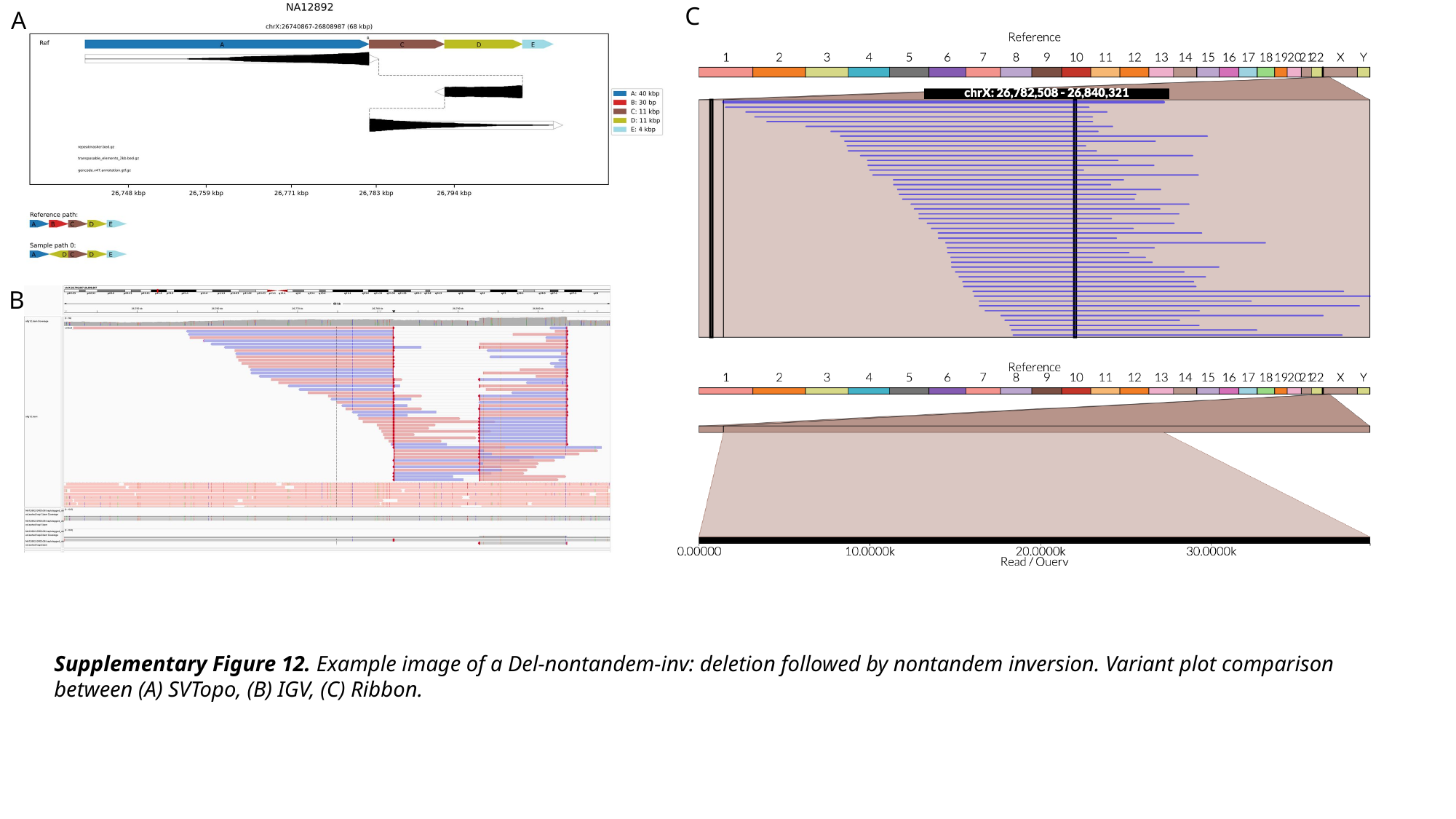

C
A
B
Supplementary Figure 12. Example image of a Del-nontandem-inv: deletion followed by nontandem inversion. Variant plot comparison between (A) SVTopo, (B) IGV, (C) Ribbon.
